# Neurodevelopmental outcomes in children with transient hypothyroxinemia of prematurity assessed using the Denver Developmental Screening Test II (DDST-II)

**DOI:** 10.20945/2359-4292-2026-0091

**Published:** 2026-08-12

**Authors:** Yavuz Özer, Aslan Yılmaz, Gizem Yılmaz, Aysun Ayaz Sarı, Hande Turan, Gürkan Tarçın, Dilek Bingöl Aydın, Ersin Ulu, Yıldız Perk, Mehmet Vural, Olcay Evliyaoğlu

**Affiliations:** 1 Istanbul University-Cerrahpasa, Cerrahpasa Medical Faculty, Department of Pediatric Endocrinology, Istanbul, Turkey; 2 Istanbul University-Cerrahpasa, Cerrahpasa Medical Faculty, Department of Pediatric Neonatology, Istanbul, Turkey; 3 Istanbul University-Cerrahpasa, Cerrahpasa Medical Faculty, Department of Pediatrics, Istanbul, Turkey; 4 Istanbul University-Cerrahpasa, Cerrahpasa Medical Faculty, Department of Pediatric Neurology, Istanbul, Turkey

**Keywords:** Transient hypothyroxinemia of prematurity: preterm infants, levothyroxine supplementation, neurodevelopment, Denver Developmental Screening Test II (DDST-II)

## Abstract

**Objective:**

This study aimed to evaluate the impact of levothyroxine supplementation on neurodevelopmental outcomes in infants with transient hypothyroxinemia of prematurity (THOP).

**Subjects and methods:**

In this retrospective observational study, infants born at < 34 weeks of gestation were categorized into three groups: THOP treated with levothyroxine, untreated THOP, and non-hypothyroxinemic controls. Neurodevelopmental outcomes were assessed at 12–72 months of corrected age using the Denver Developmental Screening Test II (DDST-II).

**Results:**

Fifty-four infants (40.7% female) with a median gestational age (GA) of 31.0 (28.6–33.0) weeks and mean birth weight of 1414 ± 466 g were included. Infants treated for THOP had significantly lower GA compared with controls (p = 0.037) and lower initial FT4 levels (p < 0.001), while TSH levels were similar (p = 0.581). Prematurity-related morbidities were more frequent in the treated THOP group. No association was observed between DDST-II results and GA, birth weight, or prematurity-related morbidities. The DDST-II results did not differ significantly among treated THOP, untreated THOP, and controls (p = 0.484).

**Conclusion:**

Levothyroxine supplementation in infants with THOP was not associated with neurodevelopmental outcomes compared with untreated infants with THOP or non-hypothyroxinemic controls. In this cohort, DDST-II results were not significantly associated with GA, birth weight, or major prematurity-related morbidities, suggesting that routine levothyroxine supplementation may not confer a measurable neurodevelopmental benefit. Given the observational design and baseline clinical differences between groups, these findings should be interpreted cautiously. Further multicenter studies with larger cohorts and comprehensive follow-up are needed to clarify whether specific high-risk subgroups may benefit from treatment..

## INTRODUCTION

Thyroid hormones (TH) are essential for normal brain development, regulating neuronal migration, myelination, synaptogenesis, and axonal growth during fetal and early postnatal life. In early gestation, the fetus depends entirely on maternal TH supply, whereas from mid-gestation onward, the hypothalamic–pituitary–thyroid (HPT) axis gradually matures and begins to contribute to hormone production (^1^,^2^). In preterm infants, particularly those born before 30 weeks, the postnatal rise in TH levels is often delayed, and normalization may take several weeks (^2^).

Transient hypothyroxinemia of prematurity (THOP), characterized by low free thyroxine (FT4) levels with normal or low thyroid-stimulating hormone (TSH) levels, is the most common thyroid dysfunction in preterm infants, affecting up to 20% of those born ≤ 34 weeks. Its multifactorial etiology includes immaturity of the HPT axis, abrupt cessation of maternal TH transfer, limited thyroidal reserve, illness-related alterations in TH metabolism, and effects of medication such as dopamine and glucocorticoids (^1^–^3^). Risk factors include low birth weight, small for gestational age (GA), and major complications of prematurity such as respiratory distress syndrome (RDS), chronic lung disease (CLD), intraventricular hemorrhage (IVH), necrotizing enterocolitis (NEC), patent ductus arteriosus (PDA), and sepsis (^1^–^5^).

The clinical significance of THOP remains debated. Some studies have reported an association between low neonatal TH levels and adverse neurodevelopmental outcomes (^6^–^8^), whereas others found no significant relationship (^9^–^11^). Randomized controlled trials of levothyroxine supplementation have likewise yielded conflicting results — with beneficial effects reported in extremely preterm infants (< 28 weeks) (^12^), neutral results in moderately preterm infants (^13^), and even potential harm in those born > 29 weeks (^14^). These controversies make the decision to treat THOP challenging, underscoring the need to distinguish infants who might benefit from supplementation from those in whom low FT4 represents a transient, adaptive phenomenon (^15^,^16^).

The Denver Developmental Screening Test II (DDST-II) is a practical screening tool for early detection of developmental delay in children up to 6 years of age and is widely applied in the follow-up of high-risk preterm infants (^17^). This study aimed to evaluate the neurodevelopmental outcomes of preterm infants with THOP, comparing levothyroxine-treated and untreated infants with non-hypothyroxinemic controls, while accounting for GA and prematurity-related morbidities.

## SUBJECTS AND METHODS

### Study design and participants

This single-center retrospective observational study included infants born between 24 and 34 weeks of gestation who were admitted to the neonatal intensive care unit of a tertiary care hospital between January 2014 and December 2019. Infants with chromosomal anomalies, major congenital malformations, or hypoxic–ischemic encephalopathy were excluded. Participants were categorized into three groups: (1) THOP treated with levothyroxine, (2) untreated THOP, and (3) controls without hypothyroxinemia. Neurodevelopmental outcomes were assessed at 12–72 months of corrected age using the DDST-II.

### Data collection

Perinatal and neonatal data were retrieved from electronic medical records and included GA, birth weight, Apgar scores, mode of delivery, and major complications of prematurity such as RDS, CLD, IVH, NEC, PDA, and retinopathy of prematurity (ROP). Neurodevelopmental evaluation using DDST-II was performed at follow-up visits by trained clinicians as part of routine developmental screening in our center.

### Thyroid function testing and definition of transient hypothyroxinemia of prematurity

Serum FT4 and TSH levels were measured routinely on postnatal days 7, 14, and 28 using an electrochemiluminescence immunoassay (Elecsys 2010 analyzer; Roche Diagnostics, Indianapolis, IN, USA). Because TH concentrations vary with GA in preterm infants, FT4 values were interpreted according to gestational age–specific percentile reference reported by Williams and cols. (^18^). The criteria for THOP included FT4 levels below the 10th percentile for GA with normal TSH values in at least two consecutive measurements. The decision to initiate levothyroxine therapy was made by the attending neonatologist and pediatric endocrinologist based on clinical judgment, considering FT4 levels, persistence of hypothyroxinemia, GA, and prematurity-related complications.

### Denver developmental screening test

The DDST-II is a standardized tool designed to identify developmental issues in children aged 0–72 months, facilitating early intervention. During the study period, trained and experienced healthcare professionals administered the latest standardized version of DDST-II for Turkey to all children. The DDST-II comprises 134 developmental items categorized into four domains: personal-social, fine motor, language, and gross motor skills. Upon determining the child’s chronological age, a developmental age line is established to assess developmental skill alignment. We evaluate test items based on their pass, fail, not applicable, or refusal status. We categorize test results as “normal”, “abnormal”, or “suspect”. Passing all age-appropriate items or receiving at most one caution item is considered “normal”. “Suspect” refers to receiving only one delay, two or more caution items without delay, or one delay with one or more caution items. Two or more delay items indicate an “abnormal” result (^17^).

### Statistical analysis

Statistical analyses were performed using SPSS version 21.0 (IBM Corp., Armonk, NY, USA) and Jamovi version 2.3.21. Normality of continuous variables was assessed using the Kolmogorov–Smirnov and Shapiro–Wilk tests. Normally distributed variables were expressed as mean ± standard deviation (SD) and compared among the three groups using one-way analysis of variance (ANOVA). Non-normally distributed variables were expressed as median (interquartile range [IQR]) and compared using the Kruskal–Wallis test. The DDST-II results were analyzed as categorical variables (normal, suspicious, abnormal). Categorical variables were presented as n (%) and compared using the chi-square test or Fisher’s exact test when appropriate. When a significant difference was observed in the overall three-group comparison, descriptive pairwise comparisons were performed using the Dwass–Steel–Critchlow–Fligner test to identify which group(s) contributed most to the difference. Effect sizes for categorical variables were reported as Cramer’s V correlations between DDST-II results, and clinical variables were assessed using Spearman’s rank correlation coefficient. Secondary analyses were conducted to examine the association between GA, thyroid function tests, and prematurity-related morbidities (RDS, IVH, PDA, CLD) with abnormal DDST-II results. A two-sided p value < 0.05 was considered statistically significant.

## RESULTS

A total of 54 preterm infants (22 females, 40.7%) were evaluated at a median corrected age of 2.3 years (IQR 1.8–3.4 years). Thirty-one infants had THOP, of whom 22 (71%) received levothyroxine supplementation. Twenty-three infants with normal thyroid function served as controls.

The median corrected ages were 3.2 (IQR 3.2–3.5) years in the treated THOP group, 2.2 (IQR 1.7–2.9) years in the untreated THOP group, and 1.9 (IQR 1.8–3.2) years in the control group. Additionally, GA was significantly lower in the treated THOP group compared with the non-hypothyroxinemic control group (30.4 [28.0–31.8] weeks vs. 32.3 [28.6–33.0] weeks, respectively, p = 0.037), whereas birth weight, sex distribution, and anthropometric parameters at follow-up were similar across groups (all p > 0.05). Initial TSH values did not differ (p = 0.581), but initial FT4 levels were lowest in the treated THOP group (0.77 [0.70–1.01] ng/dL), intermediate in untreated THOP (0.92 [0.74–1.07] ng/dL), and highest in controls (1.60 [1.44–1.72] ng/dL; p < 0.001). The median number of days to initiation of levothyroxine treatment was 20 (IQR 10–51). Detailed demographic, clinical, and laboratory findings are shown in **Table 1**. Infants treated with levothyroxine had lower GA and lower initial FT4 levels and exhibited a higher burden of prematurity-related morbidities compared with controls.

**Table 1.** Demographic, clinical, and laboratory characteristics of preterm infants with transient hypothyroxinemia of prematurity and controls

		Total (n=54)	Controls (n=23)	THOP Treated (n=22)	THOP Untreated (n=9)	*P* value
Age at follow-up (years)^†^		2.3 (1.8–3.4)	1.9 (1.8–3.2)	3.2 (3.2–3.5)	2.2 (1.7–2.9)	0.19*
Sex, n (%)	Female	22 (40.7)	11 (47.8)	8 (36.4)	3 (33.3)	0.65**
	Male	32 (59.3)	12 (52.2)	14 (63.6)	6 (66.7)	
Height SDS at follow-up^††^		-0.95 ± 1.20	-0.96 ± 1.19	-0.28 ± 1.79	-1.37 ± 0.61	0.11***
Weight SDS at follow-up^††^		-0.63 ± 1.72	-0.69 ± 1.58	-0.33 ± 2.15	-1.09 ± 1.72	0.92***
BMI SDS at follow-up^††^		0.12 ± 1.40	-0.14 ± 1.38	0.90 ± 1.61	0.17 ± 1.31	0.96***
Mode of delivery, n (%)	Vaginal	9 (16.7)	3 (13)	5 (22.7)	1 (11.1)	0.61**
	Cesarean	45 (83.3)	20 (87)	17 (77.3)	8 (88.9)	
Gestational age at birth (weeks)^†^		31.0 (28.6–33.0)	32.3 (28.6–33.0)	30.4 (28.0–31.8)	30.0 (28.0–31.8)	0.026* 0.037^‡^ (treated vs. controls)
Gestational age, n (%)						0.42**
24–27 weeks		11	3	6	2	
28–30 weeks		15	5	6	4	
31–34 weeks		28	15	10	3	
Birth weight (grams)^††^		1414 ± 466	1525 ± 511	1317 ± 358	1358 ± 553	0.31***
Initial serum TSH (mIU/L)^†^		4.38 (3.01–6.31)	4.46 (3.19–5.88)	5.05 (3.32–7.03)	3.66 (2.09–5.82)	0.58*
Initial serum FT4 (ng/dL)^†^		1.07 (0.77–1.55)	1.60 (1.44–1.72)	0.77 (0.70–1.01)	0.92 (0.74–1.07)	<0.001* <0.001^‡^ (treated vs. controls) <0.001^‡^ (untreated vs. controls)
Apgar scores at 1 minute^†^		4 (3–6)	6 (4–7)	4 (2.25–4.75)	4 (4–5)	0.010* 0.012^‡^ (treated vs. controls)
Apgar scores at 5 minutes^†^		7 (5–8)	7.5 (7–8)	5 (5–7)	6 (6–7)	0.007* 0.012^‡^ (treated vs. controls) 0.050^‡^ (untreated vs. controls)
IVH, n (%)		9 (16.7)	0 (0)	5 (22.7)	4 (44.4)	0.021**
RDS, n (%)		30 (55.6)	8 (34.8)	14 (63.6)	8 (88.9)	0.019**
PDA, n (%)		12 (22.2)	2 (8.7)	5 (22.7)	5 (55.6)	0.016**
ROP, n (%)		11 (20.4)	2 (8.7)	4 (18.2)	5 (55.6)	0.012**
CLD, n (%)		22 (40.7)	4 (17.4)	13 (59.1)	5 (55.6)	0.011**
NEC, n (%)		2 (3.7)	0 (0)	1 (4.5)	1 (11.1)	0.16**
Current serum FT4 (ng/dL)^†^		1.38 (1.29–1.48)	1.43 (1.30–1.50)	1.34 (1.33–1.40)	1.33 (1.24–1.43)	0.80*
Current serum TSH (mIU/L)^†^		2.40 (1.60–3.35)	2.19 (1.54–2.82)	2.14 (1.67–2.85)	2.42 (2.03–3.99)	0.60*

* Kruskal–Wallis test;

** Fisher’s exact test;

*** One-way analysis of variance (ANOVA);

‡ *post hoc* pairwise comparison (Dwass–Steel–Critchlow–Fligner test);

† Values represent median (25th–75th percentile);

†† Values represent mean ± standard deviation.

THOP: transient hypothyroxinemia of prematurity; SDS: standard deviation scores; BMI: body mass index; TSH: thyroid-stimulating hormone; FT4: free thyroxine; IVH: intraventricular hemorrhage; RDS: respiratory distress syndrome; PDA: patent ductus arteriosus; ROP: retinopathy of prematurity; CLD: chronic lung disease; NEC: necrotizing enterocolitis.

Prematurity-related morbidities differed significantly between groups. The treated THOP group had higher complication rates compared with controls: RDS (63.6% vs. 34.8%, p = 0.019), IVH (22.7% vs. 0%, p = 0.021), PDA (22.7% vs. 8.7%, p = 0.016), CLD (59.1% vs. 17.4%, p = 0.011), and ROP (18.2% vs. 8.7%, p = 0.012). Notably, NEC was rare and did not differ significantly among groups (p = 0.318).

The DDST-II results were comparable across groups (p = 0.484, Cramer’s V = 0.179). Normal results were observed in 47.8% of controls, 31.8% of treated THOP, and 44.5% of untreated THOP infants; abnormal outcomes occurred in 34.8%, 59.1%, and 33.3% of these groups, respectively (**Table 2**). No correlation was observed between DDST-II results and perinatal or neonatal variables (p > 0.05) (**Table 3**).

**Table 2.** Denver Developmental Screening Test II (DDST-II) results in infants with transient hypothyroxinemia of prematurity and controls

	Total (n=54)	Controls (n=23)	THOP Treated (n=22)	THOP Untreated (n=9)	*P* value
Personal-social, n (%) Normal Suspicious Abnormal	34 (63) 11 (20.4) 9 (16.6)	17 (73.9) 4 (17.4) 2 (8.7)	12 (54.6) 5 (22.7) 5 (22.7)	5 (55.6) 2 (22.2) 2 (22.2)	0.65*
Fine motor-adaptive, n (%) Normal Suspicious Abnormal	36 (66.6) 9 (16.7) 9 (16.7)	17 (73.9) 4 (17.4) 2 (8.7)	15 (68.2) 2 (9.1) 5 (22.7)	4 (44.5) 3 (33.3) 2 (22.2)	0.33*
Language, n (%) Normal Suspicious Abnormal	28 (51.9) 9 (16.6) 17 (31.5)	14 (60.9) 4 (17.4) 5 (21.7)	8 (36.4) 3 (13.6) 11 (50.0)	6 (66.7) 2 (22.2) 1 (11.1)	0.18*
Gross motor, n (%) Normal Suspicious Abnormal	40 (74.1) 9 (16.7) 5 (9.2)	20 (87.0) 2 (8.7) 1 (4.3)	13 (59.1) 6 (27.3) 3 (13.6)	7 (77.8) 1 (11.1) 1 (11.1)	0.31*
Result, n (%) Normal Suspicious Abnormal	22 (40.7) 8 (14.8) 24 (44.5)	11 (47.8) 4 (17.4) 8 (34.8)	7 (31.8) 2 (9.1) 13 (59.1)	4 (44.5) 2 (22.2) 3 (33.3)	0.48*

* Chi-square test or Fisher’s exact test, when appropriate.

THOP: transient hypothyroxinemia of prematurity.

**Table 3.** Correlation between Denver Developmental Screening Test II (DDST-II) results and perinatal/neonatal variables

	rho	*P* value
Age at follow-up (years)	-0.103	0.55
Gestational age at birth (weeks)	0.170	0.22
Birth weight (grams)	0.127	0.36
Apgar scores at 1 minute	0.292	0.19
Apgar scores at 5 minutes	0.376	0.09
Initial serum TSH (mIU/L)	0.092	0.51
Initial serum FT4 (ng/dL)	0.084	0.24
IVH	-0.177	0.20
RDS	0.015	0.92
PDA	-0.109	0.43
ROP	0.047	0.78
CLD	-0.045	0.75
NEC	-0.206	0.14

TSH: thyroid-stimulating hormone; FT4: free thyroxine; IVH: intraventricular hemorrhage; RDS: respiratory distress syndrome; PDA: patent ductus arteriosus; ROP: retinopathy of prematurity; CLD: chronic lung disease; NEC: necrotizing enterocolitis.

When DDST-II results were stratified into normal, suspicious, and abnormal categories, no significant differences were observed in GA, birth weight, or the frequency of individual prematurity-related morbidities — including IVH, RDS, PDA, ROP, CLD, and NEC — across the groups (all p > 0.05) (**Table 4**). This finding indicates that abnormal DDST-II outcomes were not associated with neonatal characteristics or individual prematurity-related complications in our cohort.

**Table 4.** Comparison of perinatal characteristics and prematurity-related morbidities according to Denver Developmental Screening Test II (DDST-II) results

	Total (n=54)	Normal (n=22)	Suspicious (n=8)	Abnormal (n=24)	*P* value
Gestational age (weeks)^†^	31.0 (28.6–33.0)	31.4 (28.1–33.0)	32.4 (30.7–34.4)	30.5 (28.4–32.0)	0.41*
Birth weight (grams)^††^	1359±569	1540±575	1140±546	1266±544	0.16**
IVH, n (%)	9 (16.7)	2 (9.1)	1 (12.5)	6 (25)	0.38***
RDS, n (%)	30 (55.6)	10 (45.5)	6 (75)	14 (58.3)	0.17***
PDA, n (%)	12 (22.2)	3 (13.6)	2 (25)	7 (29.2)	0.45***
ROP, n (%)	11 (20.4)	5 (22.7)	1 (12.5)	5 (20.8)	1.00***
CLD, n (%)	22 (40.7)	6 (27.3)	5 (62.5)	11 (45.8)	0.16***
NEC, n (%)	2 (3.7)	0	0	2 (8.3)	-

* Kruskal–Wallis test;

** One-way analysis of variance (ANOVA);

*** Fisher’s exact test.

IVH: intraventricular hemorrhage; RDS: respiratory distress syndrome; PDA: patent ductus arteriosus; ROP: retinopathy of prematurity; CLD: chronic lung disease; NEC: necrotizing enterocolitis.

## DISCUSSION

In this study, we evaluated whether levothyroxine supplementation in preterm infants with THOP influences neurodevelopmental outcomes, assessed using the DDST-II between 12 and 72 months of corrected age. Our main finding was that levothyroxine treatment of infants with THOP was not associated with neurodevelopmental outcomes compared with untreated infants with THOP or non-hypothyroxinemic controls. More specifically, levothyroxine treatment was not associated with improved neurodevelopmental outcomes. Importantly, infants who received treatment represented a clinically more vulnerable subgroup, with lower GA, lower FT4 levels, and a higher burden of prematurity-related morbidities at baseline. Therefore, treatment status in this retrospective cohort likely reflects underlying illness severity rather than a causal effect of levothyroxine therapy on neurodevelopment. These results are consistent with recent evidence indicating no significant benefit of levothyroxine replacement in moderately preterm infants (^9^–^11^,^19^,^20^).

Previous studies have reported conflicting results regarding the association between THOP and neurodevelopmental outcomes. Some investigators observed that lower neonatal TH levels were associated with impaired cognitive and motor development (^6^,^8^), whereas others found no such relationship (^9^–^11^). In line with our findings, Chung and cols. (^10^) and Dilli and cols. (^9^) reported that THOP was not associated with adverse outcomes when assessed using the Bayley Scales of Infant Development Second Edition (BSID II), whereas Hollanders and cols. (^11^) demonstrated no long-term association between THOP and neurodevelopment in young adulthood.

Although the overall comparison did not reach statistical significance, the proportion of abnormal DDST-II results was numerically higher in the treated THOP group. However, this pattern likely reflects baseline clinical differences rather than a detrimental effect of levothyroxine therapy. Infants who received treatment had lower GA, lower FT4 levels, and a higher burden of prematurity-related morbidities, suggesting greater clinical vulnerability in this group. Because both illness severity and prematurity-related complications may independently influence neurodevelopment, the slightly higher proportion of abnormal DDST-II results observed in treated infants is more likely to reflect underlying illness severity rather than the effect of levothyroxine therapy itself. This interpretation is further supported by the absence of a significant association between DDST-II outcomes and TH levels or individual prematurity-related morbidities in our secondary analyses.

Our results demonstrate no association between DDST-II outcomes and GA, birth weight, or individual prematurity-related morbidities, suggesting that in our cohort, neurodevelopmental outcomes were not primarily determined by these neonatal factors or thyroid status. Notably, THOP is increasingly regarded as a consequence of immaturity and clinical instability rather than a true abnormality of the HPT axis, and it often resolves spontaneously without treatment (^10^). Some authors have even suggested that hypothyroxinemia in the context of non-thyroidal illness may represent an adaptive neuroprotective mechanism that reduces protein catabolism and oxygen consumption (^15^,^21^), raising concerns that indiscriminate levothyroxine supplementation could interfere with such adaptive processes. In contrast to some earlier studies, our secondary analyses did not demonstrate significant associations between DDST-II results and GA, birth weight, or prematurity-related morbidities such as RDS, IVH, PDA, ROP, CLD, or NEC. This may reflect limited statistical power and the use of a screening rather than a diagnostic neurodevelopmental tool. While some studies, such as the one by Lin and cols. (^22^), reported that prematurity-related morbidities were stronger predictors of adverse outcomes than thyroid function, our findings align with those by Aygün and cols. (^19^), who also found no neurodevelopmental benefit from levothyroxine replacement in moderately preterm infants, despite higher morbidity rates in treated cases. Moreover, some studies even suggest that elevated FT4 levels may correlate with worse neurodevelopment (^15^,^23^).

Our findings are consistent with several previous studies reporting no clear neurodevelopmental benefit from routine levothyroxine supplementation in moderately preterm infants. Management of THOP remains controversial, and no consensus exists regarding routine levothyroxine supplementation (^1^,^2^). Early randomized trials reported heterogeneous results. Doses ranging from 4–20 µg/kg/day administered for 2–6 weeks showed no consistent benefit on neurodevelopment (^13^,^24^–^26^). However, van Wassenaer and cols. (^27^) and Briët and cols. (^14^) demonstrated that infants born before 27 weeks’ gestation benefited from supplementation, showing higher Bayley scores at 24 months, while those born at or after 27 weeks showed no benefit or even worse outcomes. Long-term follow-up studies further highlighted gestational age–specific effects. Van Wassenaer and cols. (^16^,^28^) observed improved neuromotor outcomes up to 10.5 years in infants < 27 weeks who received levothyroxine. Our cohort consisted of infants with THOP characterized by low FT4 levels with normal TSH values according to gestational age–specific references. Therefore, infants with delayed or mild TSH elevation were not included in the study population. Neurodevelopmental outcomes and potential benefits of levothyroxine therapy may differ in preterm infants with elevated TSH levels; therefore, our findings should not be generalized to this subgroup.

More recent large-scale randomized controlled trials have yielded less encouraging results. La Gamma and cols. (^29^) reported shorter ventilation and reduced retinopathy rates with supplementation in < 28-week infants, but subsequent analyses from the same cohort found no difference in neurodevelopmental outcomes at 36 months (^13^). The multicenter TIPIT trial also failed to demonstrate improvements in growth or early neurodevelopment (^30^), although a later follow-up suggested possible cognitive benefits at 42 months (^12^). Taken together, these findings indicate that levothyroxine supplementation may confer benefit primarily in the most immature infants (< 27–28 weeks), but routine treatment of all preterm infants with THOP is not supported.

Importantly, levothyroxine supplementation is not without risk. Potential adverse effects include iatrogenic hyperthyroidism and late-onset circulatory collapse in very low birth weight infants (^31^). These risks reinforce the need for careful candidate selection rather than universal treatment.

Strengths of our study include the inclusion of both treated and untreated THOP infants, along with controls, and the integration of prematurity-related morbidities into the analysis. This design allowed us to examine the potential impact of levothyroxine supplementation while accounting for important neonatal clinical factors.

Several methodological limitations should be considered when interpreting the findings of this study. First, the retrospective design limits the ability to establish causal relationships and may introduce selection bias. In addition, treatment decisions were based on clinical judgment rather than randomization, which introduces the possibility of confounding by indication. Second, the relatively small sample size, particularly in the untreated THOP group, may have limited the statistical power to detect small but clinically meaningful differences in neurodevelopmental outcomes. Third, the higher median age at follow-up in the treated THOP group may have increased the likelihood of detecting developmental delay compared with the younger control group. Because developmental screening tools are age-dependent, this difference may have influenced the detection of developmental delays and should be considered when interpreting group comparisons. Finally, neurodevelopment was assessed using the DDST-II, which is a screening tool rather than a comprehensive diagnostic assessment. Neurodevelopmental assessment was performed over a relatively wide age range (12–72 months), which may have introduced heterogeneity and potentially obscured subtle developmental differences. Nevertheless, previous studies have reported that DDST-II demonstrates good sensitivity for detecting developmental problems in preterm infants when applied after the corrected age of 12 months (^32^). More comprehensive neurodevelopmental assessments, such as standardized cognitive or intelligence tests, may be required to detect subtle developmental differences that screening tools might miss.

In conclusion, levothyroxine supplementation in moderately preterm infants with THOP was not associated with neurodevelopmental outcomes in this cohort. Infants who received treatment had lower GA, lower FT4 levels, and a higher burden of prematurity-related complications, which may partly explain the lower proportion of normal DDST-II results observed in this group. Therefore, the observed differences likely reflect underlying clinical severity rather than a direct effect of levothyroxine therapy. These findings suggest that routine levothyroxine supplementation for moderately preterm infants with THOP may not provide measurable neurodevelopmental benefit.

## Data Availability

the datasets used and/or analyzed during the current study are available from the corresponding author upon reasonable request.

## References

[1] Eerdekens A, Langouche L, Van den Berghe G, Verhaeghe J, Naulaers G, Vanhole C (2019). Review shows that thyroid hormone substitution could benefit
transient hypothyroxinaemia of prematurity but treatment strategies need to
be clarified. Acta Paediatr..

[2] Iijima S (2019). Current knowledge of transient hypothyroxinemia of prematurity:
to treat or not to treat?. J Matern Fetal Neonatal Med..

[3] Williams F, Hume R (2011). The measurement, definition, aetiology and clinical consequences
of neonatal transient hypothyroxinaemia. Ann Clin Biochem..

[4] Yilmaz A, Ozer Y, Kaya N, Turan H, Acar HC, Ercan O (2021). The factors associated with transient hypothyroxinemia of
prematurity. BMC Pediatr..

[5] Yilmaz A, Ozer Y, Kaya N, Cakir AD, Culpan HC, Perk Y (2023). Clinical indicators that influence a clinician’s decision to
start L-thyroxine treatment in prematurity with transient
hypothyroxinemia. Ital J Pediatr..

[6] Delahunty C, Falconer S, Hume R, Jackson L, Midgley P, Mirfield M, Scottish Preterm Thyroid Group (2010). Levels of neonatal thyroid hormone in preterm infants and
neurodevelopmental outcome at 5 1/2 years: millennium cohort
study. J Clin Endocrinol Metab..

[7] Ares S, Quero J, Diez J, Morreale de Escobar G (2011). Neurodevelopment of preterm infants born at 28 to 36 weeks of
gestational age: the role of hypothyroxinemia and long-term outcome at 4
years. J Pediatr Endocrinol Metab..

[8] Coquelet S, Deforge H, Hascoët JM (5). Thyroxine Threshold Is Linked to Impaired Outcomes in Preterm
Infants. Front Pediatr. 2020 May.

[9] Dilli D, Eras Z, Andiran N, Dilmen U, Sakrucu ED (2012). Neurodevelopmental evaluation of very low birth weight infants
with transient hypothyroxinemia at corrected age of 18-24
months. Indian Pediatr..

[10] Chung ML, Yoo HW, Kim KS, Lee BS, Pi SY, Lim G (2013). Thyroid dysfunctions of prematurity and their impacts on
neurodevelopmental outcome. J Pediatr Endocrinol Metab..

[11] Hollanders JJ, Israëls J, van der Pal SM, Verkerk PH, Rotteveel J, Finken MJ, Dutch POPS-19 Collaborative Study Group (2015). No Association Between Transient Hypothyroxinemia of Prematurity
and Neurodevelopmental Outcome in Young Adulthood. J Clin Endocrinol Metab..

[12] Ng SM, Turner MA, Weindling AM (2020). Neurodevelopmental Outcomes at 42 Months After Thyroxine
Supplementation in Infants Below 28 Weeks’ Gestation: A Randomized
Controlled Trial. Thyroid..

[13] van Wassenaer-Leemhuis A, Ares S, Golombek S, Kok J, Paneth N, Kase J (2014). Thyroid hormone supplementation in preterm infants born before 28
weeks gestational age and neurodevelopmental outcome at age 36
months. Thyroid..

[14] Briët JM, van Wassenaer AG, Dekker FW, de Vijlder JJ, van Baar A, Kok JH (2001). Neonatal thyroxine supplementation in very preterm children:
developmental outcome evaluated at early school age. Pediatrics..

[15] Goel D, Luig M, Maheshwari R, D’Cruz D, Goyen TA (2020). General Movement assessment and neurodevelopmental trajectory in
extremely preterm infants with hypothyroxinaemia of prematurity
(THOP). Early Hum Dev..

[16] van Wassenaer AG, Westera J, Houtzager BA, Kok JH (2005). Ten-year follow-up of children born at <30 weeks’ gestational
age supplemented with thyroxine in the neonatal period in a randomized,
controlled trial. Pediatrics.

[17] Yalaz K, Anlar B, Bayoğlu B (2010). Türk çocuklarına Denver II gelişimsel tarama
testi : el kitabı. Denver II Developmental Screening Test
Handbook.

[18] Williams FL, Simpson J, Delahunty C, Ogston SA, Bongers-Schokking JJ, Murphy N, Collaboration from the Scottish Preterm Thyroid Group (2004). Developmental trends in cord and postpartum serum thyroid
hormones in preterm infants. J Clin Endocrinol Metab..

[19] Aygün E, Yilmaz Semerci S, Çakıl Sağlık A, Yurdakul Ertürk E (2024). Neurodevelopmental Outcome of Infants with Transient
Hypothyroxinemia of Prematurity in a Newborn Intensive Care
Unit. J Clin Res Pediatr Endocrinol..

[20] Yoon SA, Chang YS, Yang M, Ahn SY, Sung SI, Cho HS (2022). Effect of levothyroxine supplementation in extremely low birth
weight infants with transient hypothyroxinemia of
prematurity. Sci Rep..

[21] Tan LO, Tan MG, Poon WB (2019). Lack of association between hypothyroxinemia of prematurity and
transient thyroid abnormalities with adverse long term neurodevelopmental
outcome in very low birth weight infants. PLoS One.

[22] Lin YC, Wang CY, Pan YW, Chen YJ, Yu WH, Chou YY (2021). Postnatal Serum Total Thyroxine of Very Preterm Infants and
Long-Term Neurodevelopmental Outcome. Nutrients..

[23] Scratch SE, Hunt RW, Thompson DK, Ahmadzai ZM, Doyle LW, Inder TE (2014). Free thyroxine levels after very preterm birth and
neurodevelopmental outcomes at age 7 years. Pediatrics.

[24] Uchiyama A, Kushima R, Watanabe T, Kusuda S (2017). Effect of L-thyroxine supplementation on very low birth weight
infants with transient hypothyroxinemia of prematurity at 3 years of
age. J Perinatol..

[25] Chowdhry P, Scanlon JW, Auerbach R, Abbassi V (1984). Results of controlled double-blind study of thyroid replacement
in very low-birth-weight premature infants with
hypothyroxinemia. Pediatrics..

[26] Vanhole C, Aerssens P, Naulaers G, Casneuf A, Devlieger H, Van den Berghe G (1997). L-thyroxine treatment of preterm newborns: clinical and endocrine
effects. Pediatr Res..

[27] van Wassenaer AG, Kok JH, de Vijlder JJ, Briët JM, Smit BJ, Tamminga P (1997). Effects of thyroxine supplementation on neurologic development in
infants born at less than 30 weeks’ gestation. N Engl J Med..

[28] van Wassenaer AG, Briët JM, van Baar A, Smit BJ, Tamminga P, de Vijlder JJ (2002). Free thyroxine levels during the first weeks of life and
neurodevelopmental outcome until the age of 5 years in very preterm
infants. Pediatrics..

[29] La Gamma EF, van Wassenaer AG, Ares S, Golombek SG, Kok JH, Quero J (2009). Phase 1 trial of 4 thyroid hormone regimens for transient
hypothyroxinemia in neonates of <28 weeks’ gestation. Pediatrics.

[30] Ng SM, Turner MA, Gamble C, Didi M, Victor S, Manning D (2013). An explanatory randomised placebo controlled trial of
levothyroxine supplementation for babies born <28 weeks’ gestation:
results of the TIPIT trial. Trials.

[31] Kawai M, Kusuda S, Cho K, Horikawa R, Takizawa F, Ono M, Hattori T, Oshiro M (2012). Nationwide surveillance of circulatory collapse associated with
levothyroxine administration in very-low-birthweight infants in
Japan. Pediatr Int..

[32] Jeong SU, Kim GC, Jeong HJ, Kim DK, Hong YR, Kim HD (2017). The Validity of the Bayley-III and DDST-II in Preterm Infants
With Neurodevelopmental Impairment: A Pilot Study. Ann Rehabil Med..

